# Implication of plasma intermedin levels in patients who underwent first-time diagnostic coronary angiography: a single centre, cross-sectional study

**DOI:** 10.1186/1471-2261-14-182

**Published:** 2014-12-11

**Authors:** Aylin Hatice Yamac, Ahmet Bacaksiz, Ziya Ismailoglu, Sitki Kucukbuzcu, Emrah Sevgili, Emin Asoglu, Muharrem Nasifov, Parviz Jafarov, Ercan Erdogan, Omer Goktekin

**Affiliations:** Faculty of Medicine, Department of Cardiology, BezmiÂlem Foundation University, Adnan Menderes Avenue, Vatan Street, 34093 Fatih, Istanbul, Turkey

**Keywords:** Intermedin, Coronary artery disease, Diagnostic coronary angiography

## Abstract

**Background:**

Intermedin (IMD) is involved in the prevention of atherosclerotic plaque progression, possessing cardioprotective effects from hypertrophy, fibrosis and ischemia-reperfusion injury. Elevated plasma IMD levels have been demonstrated in patients with acute coronary syndromes. No human study has examined the role of IMD in stable patients who underwent diagnostic coronary angiography with suspicion of coronary artery disease (CAD). Thus we investigated the role of IMD as a biomarker to discriminate patients with CAD and predict those with severe disease who require early and intensive therapeutic intervention before presenting with acute coronary syndrome.

**Methods:**

Eligible two hundred and thirty-eight consecutive patients (123 males, mean age 58.4 ± 10.0 years) who underwent first-time diagnostic coronary angiography were included in this study. Plasma concentrations of IMD were measured from arterial blood samples by the enzyme-linked immunosorbent assay. Patients were divided into three groups according to the presence and degree of CAD, consisting of 48 patients with normal coronary anatomy (Group 1), 111 patients with < 50% coronary stenosis (Group 2), and 79 patients with ≥ 50% stenosis in at least one of the major coronary arteries (group 3). The severity and extent of CAD was evaluated by calculations of the vessel, Gensini, and SYNTAX scores.

**Results:**

Circulating plasma IMD levels in patients with CAD were significantly higher than those in patients without CAD (157.7 ± 9.6, 134.8 ± 11.9, and 117.6 ± 7.9 pg/mL in groups 3, 2 and 1 respectively; *p* < 0.001). Besides, plasma IMD levels were correlated with Gensini and SYNTAX scores (r_s_ = 0.742, and r_s_ = 0.296, respectively; *p* < 0.05). The presence of ≥50% coronary artery stenosis could be predicted if a cut-off value of 147.7 pg/mL for plasma IMD was used with 88.6% sensitivity and 88.7% specificity. Moreover, a plasma IMD level of <126.6 pg/mL could discriminate a patient with normal coronary arteries from patients with angiographically proven CAD with a sensitivity and specificity of 84.7%, and 83.3% respectively.

**Conclusions:**

We demonstrated that IMD might be used as a biomarker to predict CAD and its severity in patients who underwent first time diagnostic coronary angiography.

## Background

Cardiovascular disease is the leading cause of morbidity and mortality worldwide [1]. Although atherosclerosis is the underlying pathology in general, there are many mechanisms proposed to explain the development of the disease from plaque formation to thrombus related occlusion of the artery such as, inflammation, oxidative stress, and endothelial dysfunction [2, 3]. Identification and quantification of atherosclerotic involvement is of high importance to determine patients at high risk for future cardiovascular events. Nowadays, there is considerable evidence supporting the clinical utility of several biomarkers, which are involved in the development of atherogenesis, to determine the prospective cardiovascular risk [4].

Intermedin (IMD) is a novel member of the calcitonin gene-related peptide (CGRP) family, which serves as an endocrine integrator of homeostasis in the cardiovascular and renal systems [5]. It exerts directly positive inotropic and chronotropic effects on the myocardium, besides IMD reduces the cardiac afterload via systemic and pulmonary vasodilatation, induces coronary vasodilatation, thereby enhancing myocardial perfusion [6]. In addition, it has been also reported to be cardioprotective from remodelling by inhibiting cardiac fibroblast activation induced by angiotensin II, anti-hypertrophic in conditions that promote cardiac myocyte hypertrophy, and protecting against ischemia-reperfusion injury by inhibiting formation of reactive oxygen species [5, 7, 8]. Furthermore, Zhang et al. demonstrated that exogenous administration of IMD could prevent the progression of atherosclerotic plaque formation in ApoE null mice [9]. Two recent human studies, which investigated the role of IMD in acute coronary syndromes, demonstrated that plasma levels of IMD were markedly elevated in patients with acute myocardial infarction and moreover revealed that it could be used as a marker to reflect the severity of coronary stenosis in this patient collective [10, 11].

To the best of our knowledge, there is not any data about plasma IMD concentrations and its diagnostic value in stable patients with signs and/or symptoms suggesting coronary artery disease (CAD). The aim of this study is to investigate the clinical relevance of plasma IMD levels to predict CAD in patients who underwent first-time diagnostic coronary angiography and its association with the severity and extent of the disease.

## Methods

### Study population

Patients enrolled in this study were patients who underwent diagnostic conventional coronary angiography for suspected CAD at our institution between February 1, 2013 and November 1, 2013. Exclusion criteria included histories of recent myocardial infarction (MI), unstable angina pectoris (UAP), heart failure (systolic and/or diastolic heart failure was excluded with the use of transthoracic echocardiography, left ventricular ejection fraction <40% was accepted as systolic heart failure), moderate to severe heart valve disease, malignancies, major trauma or surgery in the previous six months, renal insufficiency, acute or chronic infectious disease, chronic obstructive pulmonary disease, any kind of immune-mediated disease, since all these conditions may affect plasma IMD levels. Patients with coronary anomalies and/or slow flow phenomenon were also excluded. According to the inclusion criteria two hundred and thirty-eight eligible patients were included into the study. The local ethics committee (BezmiÂlem Foundation University Clinical Studies Ethics Committee) approved the study protocol, and all patients provided an informed consent. The study was conducted in accordance with the ethical principles described by the Declaration of Helsinki.

### Coronary angiography

Coronary angiography was performed by a monoplane cine-angiography system. Standard selective coronary angiography with at least four views of the left coronary system and two views of the right coronary artery had been performed using the Judkins technique. All angiograms were recorded at a 25 frames/second rate by using a 35 mm cinefilm and iopromide (Ultravist 370, Schering AG, Berlin, Germany) was used as opaque material. Two blinded experienced interventional cardiologists from our institute examined the angiograms. When there was a disagreement, the difference was adjusted by a third investigator.

Modified Gensini scoring system and the vessel score were used to determine the severity and extent of CAD [12, 13]. To calculate the vessel score, one point was assigned to each coronary artery when there was 50% or more stenosis in the three main coronary arteries and their major side branches [13]. One additional point was assigned when there was 50% or more stenosis in the left main coronary artery (LMCA), consequently with a total score of maximum 4. On the other hand, modified Gensini score was obtained by giving consideration to the severity of lesions at certain segments of the LMCA, left anterior descending (LAD), circumflex (Cx) and right coronary (RCA) arteries [12]. Two points were assigned for a lesion with an obstruction level between 0-24%, 4 points for a lesion of 25-49%, 8 points for a lesion of 50-74%, 16 points for a lesion of 75-89%, 32 points for a lesion of 90-99% and 64 points for a lesion of 100% obstruction. If the RCA was dominant, the lesion score for LMCA was multiplied by a constant multiplier of 5; the lesion scores for proximal LAD and Cx were multiplied by 2.5; the score for medial LAD lesion was multiplied by 1.5; the scores for RCA, distal LAD and distal Cx lesions were multiplied by 1; the scores for optus marginalis (OM1) and diagonal (D1) side branches were multiplied by 1 and the lesion score for other side branches was multiplied by 0.5. Total Gensini score was obtained by adding the resultant figures. If the left system was dominant, a constant multiplier of 0.5 multiplied the scores of proximal, medial and distal segment lesions of the RCA. Ultimately, total Gensini score was obtained numerically, indicating the severity of CAD. In addition, the Synergy between PCI with Taxus and Cardiac Surgery (SYNTAX) score was applied to all coronary lesions with a diameter stenosis greater than 50% in vessels with a dimater larger than 1.5 mm as described before [14]. Inter- and intra-observer variability for repeated evaluations of angiograms of 20 randomly selected patients was low (for Gensini score: inter-observer variability 4.1 ± 1.9%; intra-observer variability 4.4 ± 2.0%).

### Laboratory tests

Peripheral blood samples for complete blood count and serum creatinine, fasting glucose and lipid profiles were taken from an antecubital vein in the morning before the coronary angiography. All patients had normal serum creatinine levels and normal white blood cell counts. Total cholesterol, triglycerides, and high-density lipoprotein cholesterol were measured using enzymatic methods after overnight fasting. Low density lipoprotein (LDL) cholesterol concentrations were calculated using the Friedewald formula [15].

Arterial blood samples for IMD measurements were collected in EDTA anticoagulant tubes from the arterial sheath before coronary angiography. The samples were centrifuged for 10 minutes at 4°C and 3000 g within 30 minutes after collection for separation of plasma. All plasma samples were stored frozen at -40°C before use. Plasma concentrations of IMD were measured by the enzyme-linked immunosorbent assay (ELISA) kit, manufactured by MyBioSource, Inc. (San Diego, USA). The intra- and inter-assay coefficient of variation was less than 10% and 5% respectively. All procedures followed strictly the manufacturer’s instructions.

### Statistical analysis

Data were analyzed by using the SPSS software version 13.0 (SPSS, Chicago, IL). The distribution of the variables was analyzed with the Kolmogorov–Smirnow test. Continuous, normally distributed variables were presented as mean ± standart deviation (SD), and non-normally distributed variables as median (interquartile range). Categorical and ordinal data were presented as frequencies and/or percentages. Differences between groups were tested using Student’s t test or Mann–Whitney U test. Categorical variables were compared by the χ^2^-test. The Spearman’s correlation coefficient (r_s_) was computed to assess variable relations. A regression analysis was performed to detect the predictors of presence of angiographically confirmed CAD. The univariated regression model was used separately for each following covariates: age, gender, smoking status, BMI, hypertension, diabetes, dyslipidemia, and serum IMD level. The covariates, which were significantly associated with the presence of CAD in the univariated model, were included into the multivariate logistic regression analysis. A two-sided *p* value < 0.05 was considered statistically significant.

## Results

Patients’ baseline characteristics were listed in Table 1. Patients were divided into three groups according to the presence and degree of luminal stenosis on coronary angiography: 48 patients with normal coronary anatomy (group 1), 111 patients with < 50% coronary stenosis (group 2), and 79 patients with ≥ 50% stenosis in at least one of the major coronary arteries (group 3). Patients with CAD (groups 2 and 3) were mostly males and they were significantly older than patients in group 1 (*p* < 0.05). There was no significant difference in terms of BMI and waist circumference. Besides, smokers were more common in group 2, but the difference did not reach any statistical significance. However, traditional cardiovascular risk factors, such as hypertension, diabetes, and hyperlipidemia were not different in patients with and without CAD. Laboratory measures, including serum glucose, creatinine and WBC, were similar in both groups. Patients in group 3 had higher LDL-cholesterol levels compared with patients in group 1 (149.1 ± 37.6 versus 130.1 ± 28.9 mg/dL, respectively; *p* < 0.05).

**Table 1:** **Patient demographics, clinical and laboratory characteristics**

	Patients without CAD (Group 1) (n = 48)	Patients with <50% coronary stenosis (Group 2) (n = 111)	Patients with ≥50% coronary stenosis (Group 3) (n = 79)
Age (years)	52.5 ± 8.8	58.0 ± 9.9^*^	62.6 ± 9.1^*#^
Male gender (%)	14 (29.2%)	54 (48.6%)^*^	55 (69.6%)^*#^
BMI (kg/m^2^)	30.6 ± 4.6	29.0 ± 4.9	28.7 ± 5.1
Waist circumference (cm)	100.9 ± 10.2	101.4 ± 9.2	102.1 ± 8.2
Current smoker	10 (20.8%)	28 (25.2%)	26 (32.9%)
Hypertension (%)	23 (47.9%)	61 (54.9%)	34 (43.0%)
Diabetes mellitus (%)	12 (25.0%)	35 (31.5%)	24 (30.4%)
Dyslipidemia (%)	10 (20.8%)	28 (25.2%)	16 (20.2%)
SBP (mmHg)	135.8 ± 22.6	138.0 ± 22.8	137.5 ± 20.9
DBP (mmHg)	78.2 ± 8.3	79.2 ± 10.5	79.5 ± 9.3
Glucose (mg/dL)	118.2 ± 57.5	114.9 ± 31.1	117.9 ± 44.4
Creatinine (mg/dL)	0.7 ± 0.1	0.8 ± 0.2	0.8 ± 0.2
WBC (×10^3^/mL)	7.8 ± 1.8	7.4 ± 1.8	7.5 ± 1.7
Total cholesterol (mg/dL)	204.1 ± 30.5	208.7 ± 28.4	217.3 ± 40.2
LDL cholesterol (mg/dL)	130.1 ± 28.9	140.7 ± 32.0	149.2 ± 37.6^*^
HDL cholesterol (mg/dL)	41.5 ± 13.5	39.0 ± 8.7	38.4 ± 10.3
Triglyceride (mg/dL)	150.2 ± 62.1	160.5 ± 58.3	153.7 ± 55.2

BMI: Body mass index, SBP: Systolic blood pressure, DBP: Diastolic blood pressure, WBC: White blood cell count, LDL: Low density lipoprotein, HDL: High density lipoprotein.

^*^ *p* < 0.05 versus group 1.

^#^ *p* < 0.05 versus group 2.

Male patients had higher plasma IMD levels compared to females (142.3 ± 16.8 versus 135.3 ± 18.7 pg/mL, respectively; *p* < 0.01). In addition, the plasma IMD concentration was elevated in current smokers (143.4 ± 16.8 versus 137.3 ± 18.3 pg/mL, respectively; *p* = 0.02). Presence of other cardiovascular risk factors such as hypertension, diabetes mellitus, and hyperlipidemia did not affect plasma IMD levels (Table 2). A positive correlation was observed between plasma IMD levels and age (r_s_ = 0.255, *p* < 0.01).

**Table 2:** **Plasma intermedin levels according to cardiovascular risk factors**

Variable	Intermedin	***p***
Gender		
Male	142.3 ± 16.8	0.002
Female	135.3 ± 18.7	
Smoking habit		
Current smoker	143.4 ± 16.8	0.02
Nonsmokers and ex-smokers	137.3 ± 18.3	
Hypertension		
Present	138.7 ± 17.7	0.71
Absent	139.6 ± 19.0	
Diabetes mellitus		
Present	140.7 ± 16.9	0.33
Absent	138.2 ± 18.9	
Hyperlipidemia		
Present	138.1 ± 18.3	0.78
Absent	139.0 ± 19.4	

**Figure 1:**
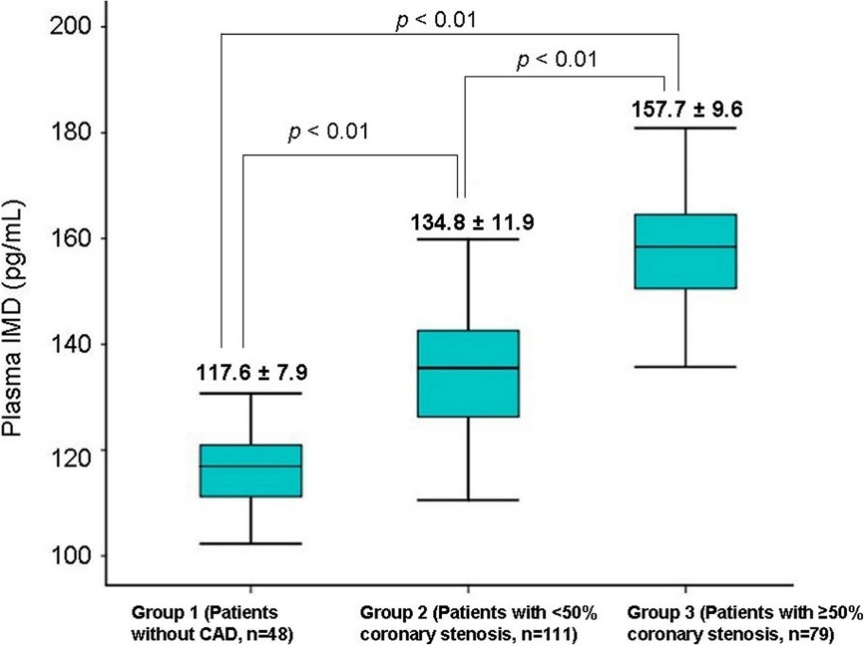
**Plasma intermedin levels among the study groups.** Data were presented as mean ± standard deviation (CAD: Coronary artery disease).

**Figure 2:**
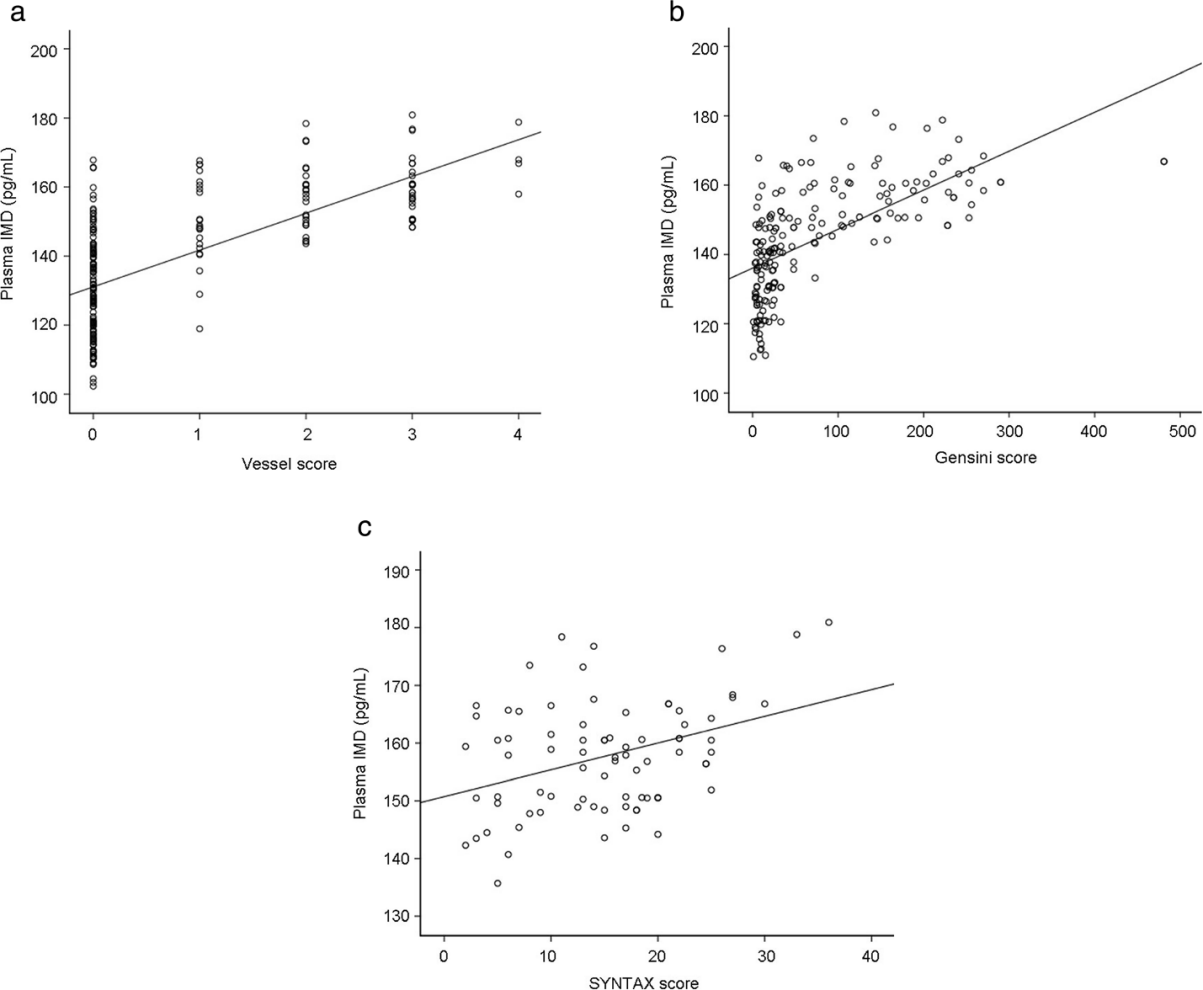
**Plasma intermedin concentration plotted against vessel (a), Gensini (b), and SYNTAX (c) scores.**

The plasma IMD concentration in patients with CAD was significantly higher than in patients without CAD (157.7 ± 9.6, 134.8 ± 11.9, and 117.6 ± 7.9 pg/mL in Groups 3, 2, and 1, respectively; *p* < 0.01) (Table 3, Figure 1). In addition, plasma IMD levels were correlated with the vessel, Gensini, and SYNYAX scores (r_s_ = 0.710, rs = 0.742, and r_s_ = 0.296, respectively; *p* < 0.01) (Figure 2a, b, and c).

**Figure 3:**
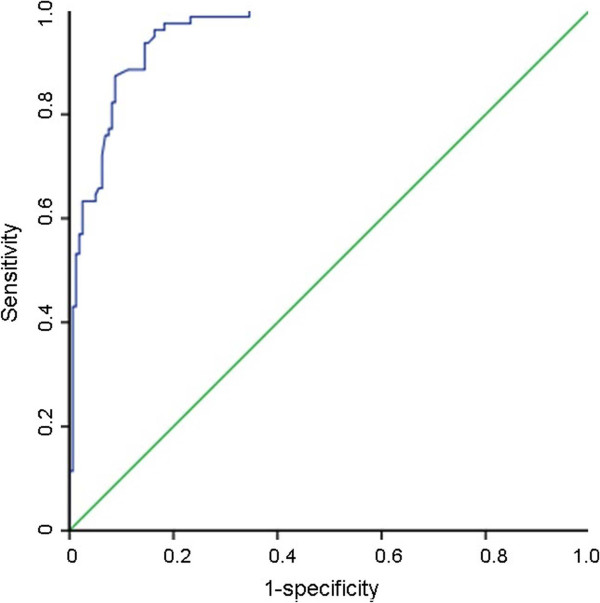
**Receiver-operating characteristic (ROC) analysis for plasma intermedin for the discrimination of patients with ≥50% stenosis at least in one of the coronary arteries from patients with normal coronary anatomy and <50% stenosis.**

ROC curve was generated for sensitivity and specificity with the respective areas under the curve (AUC) for the plasma IMD concentration. The diagnostic value for plasma IMD levels in discriminating patients with ≥ 50% coronary stenosis in at least one of the coronary arteries from those without significant CAD was high (AUC = 0.955, Figure 3). If we determined a concentration of 147.70 pg/mL as cutoff value for plasma IMD, we could predict the presence of CAD with significant stenosis with 88.6% sensitivity and 88.7% specificity. The negative predictive value was 88.7%. The diagnostic value for plasma IMD levels in discriminating patients with normal coronary arteries, who underwent coronary angiography with suspicion of CAD, was also high. A plasma IMD level of <126.60 pg/mL could discriminate a patient with normal coronary arteries from patients with angiographically proven CAD with a sensitivity and specifity of 84.7%, and 83.3% respectively.

**Table 3:** **Angiographical characteristics of the study population and plasma IMD levels of the groups**

	Patients without CAD (Group 1) (n = 48)	Patients with <50% coronary stenosis (Group 2) (n = 111)	Patients with ≥50% coronary stenosis (Group 3) (n = 79)
Gensini score	0	17.2 ± 14.3	152.2 ± 91.7
Number of diseased vessels			
0	48	50	0
1	-	-	18
2	-	-	23
3	-	-	23
4	-	-	2
Vessel score	0	0	2.2 ± 1.0
Location of stenosis (≥ 50%)			
LMCA	-	-	9
LAD	-	-	58
LCx	-	-	51
RCA	-	-	53
SYNTAX score	-	-	15.1 ± 7.8
Plasma Intermedin (pg/mL)	117.6 ± 7.9	134.8 ± 11.9^*^	157.7 ± 9.6^*,#^

LMCA: Left main coronary artery, LAD: Left coronary artery, LCx: Left circumflex artery, RCA: Right coronary artery.

^*^ *p* < 0.01 compared to group 1.

^#^ *p* < 0.01 compared to group 2.

Data were presented as mean ± standard deviation.

Multivariate linear logistic regression analysis was performed to estimate the effects of plasma IMD levels together with other cardiovascular risk factors such as age, male gender, smoking, BMI, waist circumference, hypertension, diabetes, dyslipidemia and LDL-cholesterol levels on the presence of CAD (Table 4). Plasma IMD levels were independently associated with the presence of CAD (β = 0.267, BE = 1.305, *p* < 0.001).

**Table 4:** **Multivariate logistic regression analysis of various variables (demographical features, co-morbidities and plasma intermedin levels) which determined as predictors of coronary artery disease in univariate analyses (R** ^**2**^ **= 0.63)**

Variables	β Coefficient	Odds (95% C.I.)	***p***
Current smoker	-19.60	0	1
BMI	-0.01	0.99 (0.57–1.70)	0.96
Hypertension	-0.69	0.50 (0.03–8.03)	0.62
Age	0.05	1.05 (0.89–1.23)	0.56
Dyslipidemia	-1.27	0.28 (0–20.74)	0.56
Male gender	-1.80	0.16 (0.01–2.16)	0.17
LDL-cholesterol	0.02	1.02 (0.99–1.05)	0.18
Diabetes mellitus	2.08	8.03 (0.66–98.0)	0.10
Waist circumference	- 0.10	0.90 (0.81–1.01)	0.07
Intermedin	0.27	1.30 (1.11–1.53)	<0.001

BMI: Body mass index, LDL: low-density lipoprotein.

## Discussion and conclusion

Roh and colleagues identified a new peptide that possesses similar characteristics with adrenomedullin and named it IMD due to its primarily expression in the intermediate lobe of the anterior pituitary glands [16]. This peptide is a member of the CGRP superfamily, which is well known as an endocrine and neurocrine integrator of homeostasis in the cardiovascular system [17]. Subsequent preclinical and human studies reported that IMD is also localized in the left ventricular myocytes, pericardial adipocytes, in vascular smooth muscle cells of coronary arteries, renal arterioles and tubular cells [6].

Increased expression of IMD in animal models of cardiovascular diseases such as myocardial hypertrophy, chronic hypoxia-induced pulmonary hypertension, ischemia-reperfusion injury, hypertension and heart failure suggested a key role of IMD in cardiac pathophysiology [5, 6, 18, 19]. The robust increase in expression of the peptide in hypertrophied and ischemic myocardium indicates an important protective role for IMD as an endogenous counter-regulatory peptide in the heart. This was underlined by the observation that progression of atherosclerotic plaque in ApoE null mice was terminated with exogenous administration of IMD [9].

On cell level it has been shown that intermedin protects human macrovascular, microvascular, and cardiac non-vascular cells against ischemia reperfusion injury via AM(1)-receptor signaling [20]. Furthermore, IMD exerts potent cardioprotective effects against acute rat ischemic injury [21], inhibiting endoplasmic reticulum stress via PI3 kinase-Akt signaling [22], and activating cardioprotective Akt/GSK-3beta signaling, decreasing mitochondrial-mediated myocardial apoptosis [23].

Considering the cardioprotective effects of endogenous and exogenous IMD, we assumed that plasma IMD levels might be upregulated in stable patients with suspicious CAD, countering the detrimental effects of endothelial dysfunction and oxidative stress related to CAD, even in an early phase of atherosclerotic changes.

So far, plasma levels of IMD in patients with CAD were investigated only in a few studies [10, 11]. Qin et al. demonstrated that plasma IMD levels were markedly enhanced in forty-one patients, who underwent coronary angiography with suspected acute coronary syndrome (UAP and acute MI), compared to younger healthy controls [10]. In this patient collective, high age, elevated systolic blood pressure, concentrations of brain natriuretic peptide (BNP), high-sensitivity C-reactive protein (hsCRP), creatinine kinase-MB (CK-MB), and Gensini score were correlated to plasma IMD levels. A similar study investigated the serial changes in plasma IMD levels in patients with acute ST-segment elevation MI, stable CAD patients, and healthy controls [11]. Besides the detection of higher values of plasma IMD in patients with acute MI compared to stable patients with CAD, plasma IMD concentration was gradually elevated in patients with CAD compared to healthy to controls (148.8 ± 6.0 versus 125.9 ± 9.2 pg/mL, *p* < 0.05). Significant correlations between IMD and diverse markers of oxidative stress such as superoxide dismutase and malonaldehyde during the course of acute MI supported the hypothesis that IMD might be released to protect the heart from deleterious effects related to ischemia reperfusion injury. The lack of an association between IMD levels and markers of myocardial injury such as CK-MB, Troponin-T and BNP was explained by the insensitivity of IMD for early diagnosis of MI. Thus IMD seems to be more a biomarker of a chronic process, namely atherosclerosis, helping to determine the presence and extent of CAD in a stable patient collective with suspicious CAD.

Thus our aim was to evaluate the predictive strength of IMD in selecting stable patients with CAD from healthy individuals, who were admitted to first time diagnostic angiography. We demonstrated that plasma IMD levels were enhanced in patients with angiographically confirmed CAD compared to patients with normal coronary angiograms. In addition, plasma IMD levels were significantly higher in patients with critical coronary artery stenosis (≥ 50%) than in patients with non-critical (< 50%) coronary lesions. There was a positive and significant correlation between severity and extent of CAD assessed with angiographic indexes and plasma IMD levels. Circulating levels of IMD were independently associated with the presence of CAD. Possible potential implications of this study could be regarded as follows:

- measurement of serum IMD levels could support the clinician about the presence and severity of CAD before coronary angiography.
- unnecessary coronary angiographies could be prevented by measuring serum IMD levels.
- as a biomarker of atherosclerotic progression, patients with elevated serum IMD levels could be treated more intensely, for example high dose statins, renin/angiotensin system blockers and antiplatelets. Also, close medical monitoring for the patients with elevated serum IMD levels could be reasonable to early detection and treatment of atherosclerotic involvement.

Elevated levels of IMD in patients with CAD seem to be associated with atherosclerotic development, even in patients with a subclinical disease. Besides, correlations between IMD and indices of CAD extent and severity could be regarded as increased expression of IMD in compensatory fashion to the degree of atherosclerotic involvement. Second, for the first time in the literature, our results revealed that plasma IMD levels were independently associated with the presence of CAD. Although we demonstrated significant correlations between the plasma IMD level and Gensini score similar to previous studies, IMD appeared as an independent variable associated with the presence of CAD when we evaluated the presence of CAD with other variables such as age, male gender, smoking, and LDL cholesterol in a multivariate logistic regression analysis. Furthermore, we demonstrated the essential attributes of IMD testing such as sensitivity, specifity, positive and negative predictive values for the first-time. These results suggested that IMD could be used as a biomarker to discriminate patients with CAD and predict those with severe disease who require early and intensive therapeutic intervention.

Although the correlation between serum IMD levels and angiographic indices such as vessel and Gensini score was strong (rs = 0.710 and rs = 0.742, respectively), a diminution was stood out in correlation coefficient for SYNTAX score (rs = 0.296). The SYNTAX score is a relatively new index compared to the others and primary focus on coronary anatomy, which includes defining the coronary dominance and characterizing the presence and features of chronic total occlusions, trifurcations, bifurcations, aorto-ostial lesions, severe tortuosity, long lesions, heavy calcification, thrombus and/or diffuse disease [14]. The SYNTAX score originated from the landmark Synergy between PCI with Taxus and Cardiac Surgery (SYNTAX) trial in 2009 which sought to establish whether coronary artery bypass grafting (CABG) or percutaneous coronary intervention (PCI) was the standard of care for patients with three-vessel or left main coronary artery disease. Although the score is used primarily to risk-stratify these complex patients, the use of the score for appropriate risk stratification in order to determine the optimal revascularization strategy in all CAD types are very popular nowadays. In our study, the mean SYNTAX score was very low (15.1 ± 7.8) due to a few number of patients with complex disease (for example, 2 patients with LMCA stenosis). We assume that poor correlation between SYNTAX score and serum IMD levels was resulted from relatively low SYNTAX scores and limited number of patients with high (>22) SYNTAX score in our study.

The main limitation of our study was that other markers of atherogenesis such as hsCRP, chemotactic molecules, interleukin-6 and growth differentiation factor-15 were not determined. Other pathologies that could potentially elevate serum IMD levels such as gastrointestinal, pituitary, other organ or body systems did not take into account. Also, patients were not investigated for other potential sites of atherosclerosis, such as carotid artery, thoraco-abdominal aorta, renal arteries, and peripheral vascular bed. Although potential underlying mechanisms proposed to explain the elevated plasma IMD levels in patients with CAD were discussed, the exact mechanism remains unclear. Additionally, this was a cross-sectional study and thus, the clinical impact of the results should be assessed by clinical follow-up studies.
